# Bis[2-(3-cyano­phenyl­imino­meth­yl)phenolato]nickel(II)

**DOI:** 10.1107/S1600536808004765

**Published:** 2008-02-22

**Authors:** Xing-Xuan Gong, Rong Xia, Hai-Jun Xu

**Affiliations:** a Ordered Matter Science Research Center, College of Chemistry and Chemical Engineering, Southeast UniVersity, Nanjing, 210096, People’s Republic of China

## Abstract

In the title complex, [Ni(C_14_H_9_N_2_O)_2_], the Ni^II^ atom lies on an inversion center and is coordinated by the O atom and an N atom of two Schiff base 2-(3-cyano­phenyl­imino­meth­yl)phenolate ligands in a square-planar geometry. The dihedral angle between the cyano­phenyl and phenolate rings is 47.62 (7)°.

## Related literature

For related literature, see: Adams *et al.* (2004 ▶); Bian *et al.* (2004 ▶); Brückner *et al.* (2000 ▶); Harrop *et al.* (2003 ▶); Marganian *et al.* (1995 ▶); Akkurt *et al.* (2006 ▶); Peng *et al.* (2006 ▶).
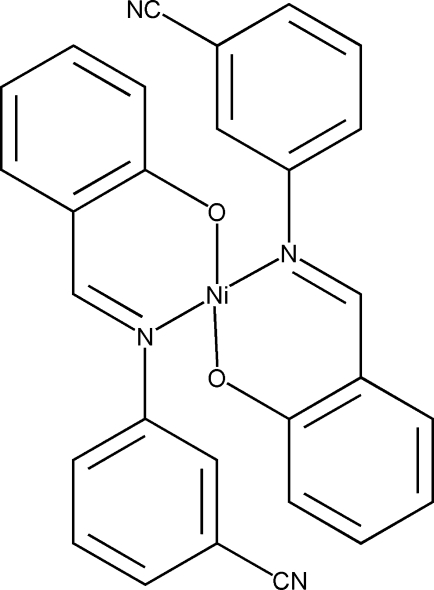


## Experimental

### 

#### Crystal data

[Ni(C_14_H_9_N_2_O)_2_]
*M* *_r_* = 501.17
Monoclinic, 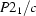

*a* = 9.0294 (18) Å
*b* = 8.0856 (16) Å
*c* = 15.644 (3) Å
β = 104.01 (3)°
*V* = 1108.1 (4) Å^3^
*Z* = 2
Mo *K*α radiation
μ = 0.91 mm^−1^
*T* = 293 (2) K
0.25 × 0.18 × 0.18 mm

#### Data collection

Rigaku Mercury2 diffractometer
Absorption correction: multi-scan (*CrystalClear*; Rigaku, 2005 ▶) *T*_min_ = 0.852, *T*_max_ = 1.00 (expected range = 0.723–0.849)
11052 measured reflections
2540 independent reflections
2246 reflections with *I* > 2σ(*I*)
*R*_int_ = 0.035

#### Refinement

*R*[*F* ^2^ > 2σ(*F* ^2^)] = 0.034
*wR*(*F* ^2^) = 0.090
*S* = 1.10
2540 reflections
160 parameters
H-atom parameters constrained
Δρ_max_ = 0.32 e Å^−3^
Δρ_min_ = −0.35 e Å^−3^

### 

Data collection: *CrystalClear* (Rigaku, 2005 ▶); cell refinement: *CrystalClear*; data reduction: *CrystalClear*; program(s) used to solve structure: *SHELXS97* (Sheldrick, 2008 ▶); program(s) used to refine structure: *SHELXL97* (Sheldrick, 2008 ▶); molecular graphics: *ORTEPIII* (Burnett & Johnson, 1996 ▶) and *ORTEP-3 for Windows* (Farrugia, 1997 ▶); software used to prepare material for publication: *SHELXL97*.

## Supplementary Material

Crystal structure: contains datablocks I, global. DOI: 10.1107/S1600536808004765/dn2310sup1.cif

Structure factors: contains datablocks I. DOI: 10.1107/S1600536808004765/dn2310Isup2.hkl

Additional supplementary materials:  crystallographic information; 3D view; checkCIF report

## Figures and Tables

**Fig. 1.**
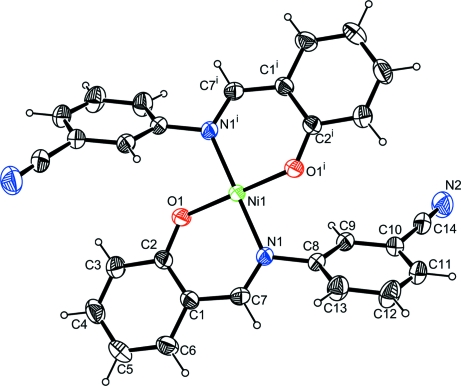
Molecular view of the title compound with the atom-labelling scheme. Displacement ellipsoids are drawn at the 50% probability level. H atoms are represented as small spheres of arbitrary radii. [Symmetry code: (i) 1 - x, 1 - y, 1 - z]

## supplementary crystallographic information

### Comment

Schiff bases have been used extensively as ligands in the field of coordination chemistry. These complexes play an important role in the development of pharmacological and catalytic properties (Harrop *et al.*, 2003; Brückner *et al.*, 2000). Nickel(II) compounds with Schiff bases have received much attention in recent years (Marganian *et al.*, 1995). Here we report the molecular and crystal structure of nickel (II) complex with a Schiff base ligand.

The Ni^II ^atom in (I) lies on an inversion center and is coordinated by the two imine N and two phenolateO atoms of the two Schiff base ligands in a square-planar geometry (Fig.1). The dihedral angle between the cyanophenyl and phenyl rings is 47.62 (7)°.. The Ni—O and Ni—N bond lengths agree with the values reported for related complexes(Peng, *et al.*, (2006); Adams *et al.*, 2004; Bian *et al.*, 2004).

### Experimental

2-(3-cyanophenyliminomethyl)phenol was prepared according to the literature (Akkurt *et al.*, 2006). NiCl_2_.6H_2_O(23.7 mg, 0.1 mmol) in methanol (5 ml) was added to the solution of 2-(3-cyanophenyliminomethyl)phenol (22.2 mg, 0.1 mmol)in the methanol (5 ml), the pH was then adjusted to 8–9 and the mixture was stirred for 4 h. The filtrate was kept at room temperature for about two weeks, and blue block shaped crystals of (I) suitable for for X-ray single-crystal analyses were obtained.

### Refinement

All H atoms attached to C atoms were fixed geometrically and treated as riding with C—H = 0.93 Å and *U*_iso_(H) = 1.2*U*_eq_(C).

### Crystal data

**Table:**

[Ni(C_14_H_9_N_2_O_1_)_2_]	*F*_000_ = 516
*M**_r_* = 501.17	*D*_x_ = 1.502 Mg m^−^^3^
Monoclinic, *P*2_1_/*c*	Mo *K*α radiation λ = 0.71073 Å
Hall symbol: -P 2ybc	Cell parameters from 10336 reflections
*a* = 9.0294 (18) Å	θ = 3.1–27.4º
*b* = 8.0856 (16) Å	µ = 0.91 mm^−^^1^
*c* = 15.644 (3) Å	*T* = 293 (2) K
β = 104.01 (3)º	Block, blue
*V* = 1108.1 (4) Å^3^	0.25 × 0.18 × 0.18 mm
*Z* = 2	

### Data collection

**Table:**

Rigaku Mercury2 diffractometer	2540 independent reflections
Radiation source: fine-focus sealed tube	2246 reflections with *I* > 2σ(*I*)
Monochromator: graphite	*R*_int_ = 0.035
Detector resolution: 13.6612 pixels mm^-1^	θ_max_ = 27.5º
*T* = 293(2) K	θ_min_ = 3.1º
ω scans	*h* = −11→11
Absorption correction: multi-scan(CrystalClear; Rigaku, 2005)	*k* = −10→10
*T*_min_ = 0.852, *T*_max_ = 1.00	*l* = −20→20
11052 measured reflections	

### Refinement

**Table:**

Refinement on *F*^2^	Secondary atom site location: difference Fourier map
Least-squares matrix: full	Hydrogen site location: inferred from neighbouring sites
*R*[*F*^2^ > 2σ(*F*^2^)] = 0.034	H-atom parameters constrained
*wR*(*F*^2^) = 0.090	*w* = 1/[σ^2^(*F*_o_^2^) + (0.0403*P*)^2^ + 0.5389*P*] where *P* = (*F*_o_^2^ + 2*F*_c_^2^)/3
*S* = 1.10	(Δ/σ)_max_ < 0.001
2540 reflections	Δρ_max_ = 0.32 e Å^−^^3^
160 parameters	Δρ_min_ = −0.35 e Å^−^^3^
Primary atom site location: structure-invariant direct methods	Extinction correction: none

### Special details

**Table:**

Geometry. All e.s.d.'s (except the e.s.d. in the dihedral angle between two l.s. planes) are estimated using the full covariance matrix. The cell e.s.d.'s are taken into account individually in the estimation of e.s.d.'s in distances, angles and torsion angles; correlations between e.s.d.'s in cell parameters are only used when they are defined by crystal symmetry. An approximate (isotropic) treatment of cell e.s.d.'s is used for estimating e.s.d.'s involving l.s. planes.
Refinement. Refinement of *F*^2^ against ALL reflections. The weighted *R*-factor *wR* and goodness of fit *S* are based on *F*^2^, conventional *R*-factors *R* are based on *F*, with *F* set to zero for negative *F*^2^. The threshold expression of *F*^2^ > σ(*F*^2^) is used only for calculating *R*-factors(gt) *etc*. and is not relevant to the choice of reflections for refinement. *R*-factors based on *F*^2^ are statistically about twice as large as those based on *F*, and *R*- factors based on ALL data will be even larger.

### Fractional atomic coordinates and isotropic or equivalent isotropic displacement parameters (Å^2^)

**Table:**

	*x*	*y*	*z*	*U*_iso_*/*U*_eq_	
Ni1	0.5000	0.5000	0.5000	0.02514 (11)	
O1	0.66446 (15)	0.36034 (17)	0.52053 (9)	0.0349 (3)	
N1	0.41172 (16)	0.38940 (18)	0.58415 (10)	0.0266 (3)	
C9	0.3215 (2)	0.6210 (2)	0.65595 (12)	0.0302 (4)	
H9	0.4159	0.6714	0.6613	0.036*	
C14	0.2380 (2)	0.8635 (3)	0.72431 (15)	0.0401 (5)	
C1	0.5678 (2)	0.1429 (2)	0.59534 (12)	0.0283 (4)	
C10	0.2087 (2)	0.6997 (2)	0.68770 (12)	0.0318 (4)	
C8	0.2929 (2)	0.4678 (2)	0.61652 (12)	0.0274 (4)	
C11	0.0679 (2)	0.6238 (3)	0.68133 (14)	0.0395 (5)	
H11	−0.0069	0.6759	0.7034	0.047*	
C3	0.7949 (2)	0.1080 (3)	0.54150 (14)	0.0383 (5)	
H3	0.8663	0.1492	0.5130	0.046*	
C2	0.6729 (2)	0.2098 (2)	0.55130 (11)	0.0286 (4)	
C13	0.1528 (2)	0.3938 (3)	0.60894 (15)	0.0404 (5)	
H13	0.1329	0.2914	0.5815	0.048*	
C7	0.4475 (2)	0.2409 (2)	0.61321 (12)	0.0297 (4)	
H7	0.3897	0.1942	0.6488	0.036*	
C5	0.7059 (3)	−0.1167 (3)	0.61697 (15)	0.0432 (5)	
H5	0.7169	−0.2245	0.6382	0.052*	
N2	0.2618 (3)	0.9944 (2)	0.75059 (17)	0.0570 (6)	
C12	0.0409 (2)	0.4716 (3)	0.64212 (17)	0.0461 (6)	
H12	−0.0527	0.4199	0.6377	0.055*	
C4	0.8094 (3)	−0.0502 (3)	0.57343 (15)	0.0430 (5)	
H4	0.8904	−0.1149	0.5658	0.052*	
C6	0.5875 (3)	−0.0197 (2)	0.62794 (14)	0.0366 (4)	
H6	0.5184	−0.0625	0.6577	0.044*	

### Atomic displacement parameters (Å^2^)

**Table:**

	*U*^11^	*U*^22^	*U*^33^	*U*^12^	*U*^13^	*U*^23^
Ni1	0.02507 (18)	0.02233 (17)	0.03093 (19)	0.00178 (12)	0.01241 (13)	0.00362 (12)
O1	0.0309 (7)	0.0327 (7)	0.0458 (8)	0.0070 (6)	0.0182 (6)	0.0125 (6)
N1	0.0262 (7)	0.0245 (7)	0.0319 (7)	−0.0009 (6)	0.0124 (6)	−0.0003 (6)
C9	0.0294 (9)	0.0286 (9)	0.0360 (9)	−0.0003 (7)	0.0146 (8)	0.0016 (7)
C14	0.0431 (12)	0.0363 (12)	0.0480 (12)	0.0067 (9)	0.0247 (10)	0.0000 (9)
C1	0.0305 (9)	0.0236 (9)	0.0309 (9)	0.0009 (7)	0.0075 (7)	0.0007 (7)
C10	0.0348 (10)	0.0305 (9)	0.0332 (9)	0.0058 (8)	0.0140 (8)	0.0022 (8)
C8	0.0282 (9)	0.0272 (9)	0.0300 (9)	0.0015 (7)	0.0133 (7)	0.0023 (7)
C11	0.0327 (10)	0.0449 (12)	0.0461 (11)	0.0093 (9)	0.0194 (9)	0.0032 (9)
C3	0.0335 (10)	0.0412 (11)	0.0425 (11)	0.0088 (9)	0.0134 (9)	0.0043 (9)
C2	0.0294 (9)	0.0282 (9)	0.0278 (9)	0.0036 (7)	0.0064 (7)	0.0020 (7)
C13	0.0348 (10)	0.0378 (11)	0.0521 (12)	−0.0068 (9)	0.0174 (9)	−0.0090 (9)
C7	0.0315 (9)	0.0263 (9)	0.0335 (9)	−0.0034 (7)	0.0122 (7)	0.0022 (7)
C5	0.0491 (13)	0.0250 (10)	0.0528 (13)	0.0073 (9)	0.0070 (10)	0.0048 (9)
N2	0.0685 (15)	0.0369 (11)	0.0762 (15)	−0.0001 (9)	0.0379 (12)	−0.0109 (10)
C12	0.0294 (10)	0.0550 (14)	0.0592 (14)	−0.0081 (9)	0.0212 (10)	−0.0084 (11)
C4	0.0407 (12)	0.0397 (11)	0.0476 (12)	0.0173 (9)	0.0085 (9)	0.0018 (10)
C6	0.0398 (11)	0.0266 (10)	0.0437 (11)	−0.0013 (8)	0.0104 (9)	0.0045 (8)

### Geometric parameters (Å, °)

**Table:**

Ni1—O1^i^	1.8310 (14)	C8—C13	1.378 (3)
Ni1—O1	1.8310 (14)	C11—C12	1.371 (3)
Ni1—N1^i^	1.9174 (15)	C11—H11	0.9300
Ni1—N1	1.9174 (15)	C3—C4	1.368 (3)
O1—C2	1.304 (2)	C3—C2	1.413 (3)
N1—C7	1.297 (2)	C3—H3	0.9300
N1—C8	1.440 (2)	C13—C12	1.393 (3)
C9—C8	1.380 (3)	C13—H13	0.9300
C9—C10	1.391 (3)	C7—H7	0.9300
C9—H9	0.9300	C5—C6	1.370 (3)
C14—N2	1.137 (3)	C5—C4	1.390 (3)
C14—C10	1.442 (3)	C5—H5	0.9300
C1—C6	1.406 (2)	C12—H12	0.9300
C1—C2	1.409 (3)	C4—H4	0.9300
C1—C7	1.426 (3)	C6—H6	0.9300
C10—C11	1.393 (3)		
			
O1^i^—Ni1—O1	180.000 (1)	C10—C11—H11	120.4
O1^i^—Ni1—N1^i^	92.65 (6)	C4—C3—C2	120.89 (19)
O1—Ni1—N1^i^	87.35 (6)	C4—C3—H3	119.6
O1^i^—Ni1—N1	87.35 (6)	C2—C3—H3	119.6
O1—Ni1—N1	92.65 (6)	O1—C2—C1	123.56 (17)
N1^i^—Ni1—N1	180.000 (1)	O1—C2—C3	118.75 (17)
C2—O1—Ni1	127.78 (12)	C1—C2—C3	117.68 (17)
C7—N1—C8	115.34 (15)	C8—C13—C12	120.4 (2)
C7—N1—Ni1	124.25 (13)	C8—C13—H13	119.8
C8—N1—Ni1	120.35 (12)	C12—C13—H13	119.8
C8—C9—C10	119.75 (18)	N1—C7—C1	125.63 (17)
C8—C9—H9	120.1	N1—C7—H7	117.2
C10—C9—H9	120.1	C1—C7—H7	117.2
N2—C14—C10	177.8 (2)	C6—C5—C4	118.5 (2)
C6—C1—C2	119.71 (18)	C6—C5—H5	120.7
C6—C1—C7	118.93 (18)	C4—C5—H5	120.7
C2—C1—C7	121.21 (16)	C11—C12—C13	120.4 (2)
C9—C10—C11	120.51 (18)	C11—C12—H12	119.8
C9—C10—C14	118.81 (18)	C13—C12—H12	119.8
C11—C10—C14	120.64 (18)	C3—C4—C5	121.6 (2)
C13—C8—C9	119.78 (17)	C3—C4—H4	119.2
C13—C8—N1	121.69 (17)	C5—C4—H4	119.2
C9—C8—N1	118.53 (16)	C5—C6—C1	121.6 (2)
C12—C11—C10	119.20 (19)	C5—C6—H6	119.2
C12—C11—H11	120.4	C1—C6—H6	119.2

Symmetry codes: (i) −*x*+1, −*y*+1, −*z*+1.
